# Enhanced skyrmion stability due to exchange frustration

**DOI:** 10.1038/s41598-017-12525-x

**Published:** 2017-09-26

**Authors:** S. von Malottki, B. Dupé, P. F. Bessarab, A. Delin, S. Heinze

**Affiliations:** 1 Institute of Theoretical Physics and Astrophysics, University of Kiel, 24098 Kiel, Germany; 2 Institute of Physics, University of Mainz, 55128 Mainz, Germany; 3 School of Engineering and Natural Sciences - Science Institute, University of Iceland, 107 Reykjavik, Iceland; 4 University ITMO, St. Petersburg, 197101 Russia; 5 Department of Applied Physics, School of Engineering Sciences, KTH Royal Institute of Technology, Electrum 229, SE-16440 Kista, Sweden; 6 Department of Physics and Astronomy, Materials Theory Division, Uppsala University, Box 516, SE-75120 Uppsala, Sweden

**Keywords:** Magnetic properties and materials, Spintronics

## Abstract

Skyrmions are localized, topologically non-trivial spin structures which have raised high hopes for future spintronic applications. A key issue is skyrmion stability with respect to annihilation into the ferromagnetic state. Energy barriers for this collapse have been calculated taking only nearest neighbor exchange interactions into account. Here, we demonstrate that exchange frustration can greatly enhance skyrmion stability. We focus on the prototypical film system Pd/Fe/Ir(111) and use an atomistic spin model parametrized from first-principles calculations. We show that energy barriers and critical fields of skyrmion collapse as well as skyrmion lifetimes are drastically enhanced due to frustrated exchange and that antiskyrmions are metastable. In contrast an effective nearest-neighbor exchange model can only account for equilibrium properties of skyrmions such as their magnetic field dependent profile or the zero temperature phase diagram. Our work shows that frustration of long range exchange interactions – a typical feature in itinerant electron magnets – is a route towards enhanced skyrmion stability even in systems with a ferromagnetic ground state.

## Introduction

Skyrmions have been predicted to occur in magnetic materials based on micromagnetic models more than 25 years ago^1,2^. Experimentally skyrmions were first observed in bulk materials with a chiral crystal structure^3–5^. In these systems skyrmions are stabilized by the Dzyaloshinskii-Moriya interaction (DMI)^6,7^. The DMI occurs due to spin-orbit coupling (SOC) in systems with a broken structural inversion symmetry. Therefore, it is present also at surfaces or interfaces^8,9^ and can induce spin spirals^9,10^, chiral domain walls^11–15^ and skyrmions^16,17^. Since transition-metal interfaces and multilayers are at the heart of technologically established spintronic devices such as read heads of hard-disk drives based on the giant magnetoresistance effect^18,19^ the discovery of skyrmions at interfaces^16,17^ sparked great interest in using them for novel device concepts^20,21^.

For potential applications^20–23^ the stability of magnetic skyrmions becomes a crucial issue. An experimentally well studied system for nanoscale skyrmions at interfaces is Pd/Fe/Ir(111)^17,24–27^, i.e. a single atomic layer of Pd grown on an Fe monolayer on the Ir(111) surface. Based on fitting field dependent experimental skyrmion profiles to the micromagnetic model effective parameters have been obtained^24^ which were used to discuss properties of isolated skyrmions^27^. Current-induced skyrmion annihilation has also been studied combining the experiments with Monte-Carlo simulations from which energy barriers of about 50 meV have been estimated^26^. Energy barriers protecting skyrmion states in ultrathin films have been obtained by calculating minimum energy paths (MEPs) for the skyrmion collapse into the ferromagnetic state^28–31^ which due to the huge configuration space is a non-trivial task^32,33^.

While an atomistic spin model has been used in these approaches, the exchange interaction has been treated within the nearest-neighbor (NN) approximation which corresponds to the exchange stiffness within the micromagnetic model. For Pd/Fe/Ir(111) it has been shown based on density functional theory (DFT) calculations that the exchange interaction is ferromagnetic for NN while it is antiferromagnetic for the 2nd and 3rd NN^34,35^. The effect of exchange frustration on skyrmion lattice formation has been studied in refs^36–39^. Therefore, it is not clear in how far spin models using an effective NN exchange interaction are sufficient to describe properties of isolated skyrmions in these systems.

Here, we demonstrate that frustrated exchange greatly enhances skyrmion stability in ultrathin films and can lead to metastable antiskyrmions. We use atomistic spin dynamics simulations parametrized from DFT for Pd/Fe/Ir(111) and obtain energy barriers for skyrmion annihilation into the ferromagnetic state by applying the geodesic nudged elastic band (GNEB) approach^28^. We compare these calculations with an effective NN exchange model based on our DFT calculations which yields parameters that are in good agreement with those obtained from experiments^24,26^. We find that equilibrium skyrmion properties such as field-dependent profiles or the zero temperature phase diagram are well described by the effective NN exchange approach. However, energy barriers of skyrmion collapse and skyrmion lifetimes are greatly underestimated if the frustrated exchange interaction is mapped to an effective NN exchange. Antiskyrmions are not even metastable in the effective NN exchange model.

## Results

### Atomistic spin model

We describe the magnetic properties of Pd/Fe/Ir(111) using the spin Hamiltonian given by

1 $$\begin{array}{c}H=-\sum _{ij}{J}_{ij}({{\bf{m}}}_{i}\cdot {{\bf{m}}}_{j})-\sum _{ij}{{\bf{D}}}_{ij}\cdot ({{\bf{m}}}_{i}\times {{\bf{m}}}_{j})\\ \quad \quad -\sum _{i}K{({m}_{i}^{z})}^{2}-\sum _{i}{\mu }_{s}{\bf{B}}\cdot {{\bf{m}}}_{i}\,,\end{array}$$

which describes the magnetic interactions between the magnetic moments **M**_*i*_ of atoms at sites **R**_*i*_ where **m**_i_ = **M**_i_/M_i_. The parameters for the exchange interaction (*J*_*ij*_), the DMI (**D**_ij_), the magnetic moments (*μ*_*s*_) as well as an uniaxial magnetocrystalline anisotropy (*K*) were obtained from DFT using the FLEUR code^34,40–42^ (see methods for details). Note that *J*_*ij*_ and **D**_ij_ are defined per spin because each pairwise interaction contributes twice in the total energy. We do not explicitly include dipole-dipole interactions. However, for ultrathin films this energy term is very small – on the order of 0.1 meV/atom – and it can be effectively included into the magnetocrystalline anisotropy energy^30,43^.

**Figure 1:**
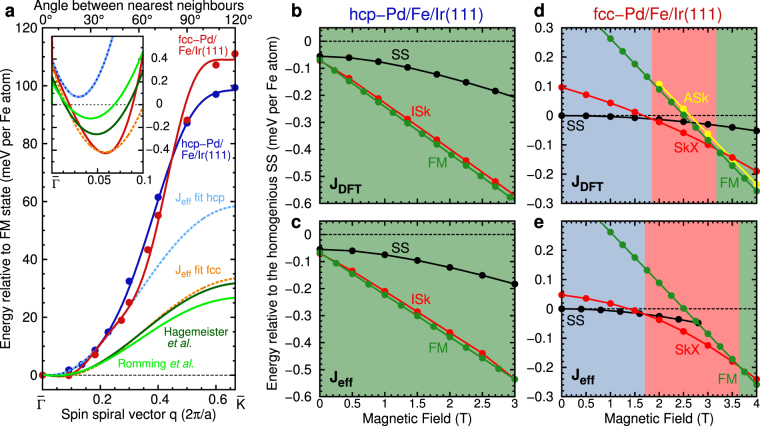
Energy dispersion of spin spirals and zero temperature phase diagrams of Pd/Fe/Ir(111). **(a)** Energy dispersion of homogeneous cycloidal right-rotating spin spirals for Pd/Fe/Ir(111) as a function of the spin spiral vector **q** along the $\bar{{\rm{\Gamma }}}-\bar{{\rm{K}}}$-direction. The inset shows a zoom of the dispersion around the energy minima. Note that there is an offset for all curves at **q** = 0 due to the magnetocrystalline anisotropy which leads to an energy increase for all spin spiral states of *K*/2 with respect to the ferromagnetic state. The filled circles are total energies obtained from DFT for fcc (red) or hcp (blue) stacking of the Pd layer including spin-orbit coupling. The solid lines are fits to the Heisenberg model including the Dzyaloshinskii-Moriya interaction. The dashed lines are fits to the DFT energy dispersion close to the energy minima with an effective nearest-neighbor exchange interaction *J*_eff_ and effective DMI for fcc (orange) and hcp (blue) stacking. For comparison, the energy dispersions for parameters given by Romming *et al*.^24^ (light green) and Hagemeister *et al*.^26^ (dark green) are shown. **(b,c)** Zero temperature phase diagram for hcp-Pd/Fe/Ir(111) obtained with the DFT (*J*_DFT_) and effective (*J*_eff_) parameters, respectively. The energies of the ferromagnetic (FM), isolated skyrmion (ISk) and relaxed spin spiral (SS) state are shown relative to the homogeneous spin spiral (dashed line). The green color indicates the regime of the FM ground state. **(d,e)** Zero temperature phase diagram for fcc-Pd/Fe/Ir(111) as in **(b,c)** including the skyrmion lattice (SkX) and the isolated antiskyrmion (ASk) state. Blue, red, and green color represents the regime of the SS, SkX, and FM ground state, respectively.

While the Fe layer follows the fcc stacking of the Ir(111) surface, both Pd stackings have been observed experimentally^44^. Figure 1a shows the energy dispersions *E*(**q**) of flat homogeneous spin spirals including the effect of SOC calculated from DFT for both hcp and fcc stacking of the Pd overlayer. A spin spiral is characterized by a wave vector **q** from the two-dimensional Brillouin zone (2D-BZ) and the magnetic moment of an atom at site **R**_i_ is given by ${{\bf{M}}}_{i}=M(\sin ({\bf{q}}{{\bf{R}}}_{i}),\,\cos ({\bf{q}}{{\bf{R}}}_{i}),\,\mathrm{0)}$ with the size of the magnetic moment *M*. We find a value of about 2.7 *μ*_B_ per Fe-atom in our calculations which is fairly constant for all **q** vectors. Combined with the magnetic moment of 0.3 *μ*_B_ due to spin polarization of the Pd-atoms, we obtain a total moment of 3.0 *μ*_B_ that we use for our simulations. Fitting the energy dispersions along the high symmetry directions of the 2D-BZ using the first two terms of Eq. (1) allows to extract the exchange constants and the DMI (see Table 1). Note that the stacking of the Pd overlayer affects the Ruderman-Kittel-Kasuya-Yosida type exchange mediated by conduction electrons due to the key role played by the hybridization at the Pd/Fe/Ir interface^34^. Our computed shell resolved *J*_*ij*_ agree well with those presented in ref.^35^. In order to obtain a good fit of the DFT energy dispersion many shells need to be taken into account for the exchange while the DMI has been treated in the NN approximation.

**Table 1:** Exchange constants, DMI and magnetocrystalline anisotropy obtained from DFT for hcp-Pd/Fe/Ir(111) and fcc-Pd/Fe/Ir(111).

	*J*_1_	*J*_2_	*J*_3_	*J*_4_	*J*_5_	*J*_6_	*J*_7_	*J*_8_	*J*_9_	*D*_1_	*K*
hcp	13.66	−0.51	−2.88	0.07	0.55	—	—	—	—	1.2	0.8
fcc	14.40	−2.48	−2.69	0.52	0.74	0.28	0.16	−0.57	−0.21	1.0	0.7

The positive sign of the DMI indicates favoring of right rotating spin spirals and the positive sign of *K* indicates an easy out-of-plane magnetization direction. The coefficients are given in meV.

It has been suggested to characterize the behavior of the exchange interaction close to the $\bar{{\rm{\Gamma }}}$ point of the 2D-BZ – corresponding to the ferromagnetic (FM) state – by an effective NN exchange constant *J*_eff_^45^ – an approach which has also been used in previous theoretical studies of skyrmion stability^26–28^.

From our DFT energy dispersion we can obtain *J*_eff_ by using only the NN terms of the first two terms in the spin Hamiltonian, Eq. (1), which yields along the $\bar{{\rm{\Gamma }}}-\bar{{\rm{K}}}$ direction $E(q)={J}_{{\rm{eff}}}(2\,\cos (q)+4\,\cos (q\mathrm{/2)})$ (with *q* in units of 2*π*/*a*, see Table 2 for *J*_eff_). We obtain an excellent approximation around the local spin spiral energy minimum (cf. inset of Fig. 1a). However, the fit drastically underestimates *E*(*q*) from DFT for larger values of *q*. For fcc-Pd/Fe/Ir(111) we had to fit also the DMI to obtain a good agreement. For spin spirals along $\bar{{\rm{\Gamma }}}-\bar{{\rm{K}}}$ we use $E(q)={J}_{{\rm{eff}}}(2\,\cos (q)+4\,\cos (q\mathrm{/2)})+{D}_{{\rm{eff}}}(2\,\sin (q)+2\,\sin (q\mathrm{/2)})$ and a fit interval close to the energy minimum (see Table 2 for *J*_eff_ and *D*_eff_).

**Table 2:** Parameters of the effective NN exchange model obtained by fitting the DFT results for hcp-Pd/Fe/Ir(111) and fcc-Pd/Fe/Ir(111) and values from the literature.

	*J*_eff_	*D*_eff_	*K*
hcp fit	6.44	1.2	0.8
fcc fit	3.68	1.39	0.7
Ref.^24^	2.95	0.8	0.4
Ref.^26^	3.5	1.1	0.5

For hcp *D*_eff_ has been chosen as the NN DFT value (cf. Table 1). Sign convention as in Table 1. The coefficients are given in meV.

Effective NN exchange and DMI for Pd/Fe/Ir(111) have previously been extracted by fitting experimental data to micromagnetic and Monte-Carlo simulations^24,26^ (for values see Table 2). The energy dispersions obtained with these values are displayed in Fig. 1a. These curves are between the DFT dispersions for hcp and fcc Pd stacking around the energy minimum (cf. inset of Fig. 1a). For larger values of *q*, i.e. larger angles between adjacent spins, they are close to the effective fit in fcc stacking but deviate drastically from the DFT curves.

### Zero temperature phase diagrams

To compare the effective description of the exchange (denoted as *J*_eff_) and DMI (for fcc-Pd/Fe/Ir(111)) and the full set of exchange constants from DFT (denoted as *J*_DFT_), we have obtained the phase diagram at zero temperature as a function of the applied magnetic field (Fig. 1b–e) using spin-dynamics simulations (see methods). Note that we have used the magnetocrystalline anisotropy obtained from DFT in both cases.

For hcp-Pd/Fe/Ir(111) the phase diagrams are very similar for both ways of treating the exchange [Fig. 1b,c]. The FM state is the ground state over the whole range of magnetic field values. Homogeneous spin spirals relax into a domain wall structure which gains energy due to DMI^11^. Isolated skyrmions are metastable for both sets of parameters when an external field of at least ≈0.5 T is applied. For smaller fields, the skyrmions become large bubbles with a fixed rotational sense of the domain walls surrounding the FM core.

For fcc-Pd/Fe/Ir(111) [Fig. 1d,e] the ground state is a spin spiral consistent with the energy minimum in the spin spiral dispersion curve (cf. inset of Fig. 1a). For both *J*_eff_ and *J*_DFT_ a skyrmion lattice is energetically favorable at a critical field and at even larger fields there is a transition to the FM phase. However, the critical fields at which these transitions occur are slightly different (${B}_{{\rm{c}}}^{{\rm{eff}}}\approx 3.6$ T and ${B}_{{\rm{c}}}^{{\rm{DFT}}}\approx 3.2$ T) due to the larger DMI obtained by the fit. Using the full set of DFT parameters we were also able to metastabilize antiskyrmions within the skyrmion lattice and FM phase.

### Skyrmion radius

**Figure 2:**
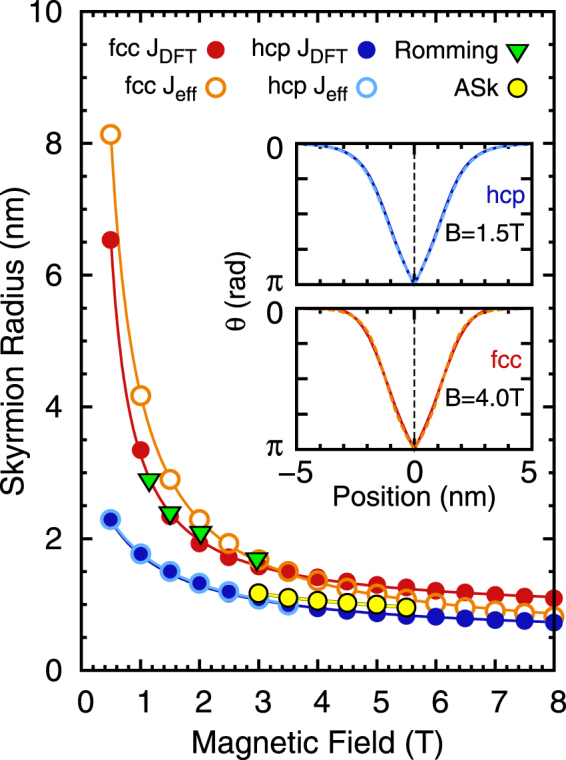
Skyrmion radius vs. magnetic field. Radii of skyrmions in Pd/Fe/Ir(111) obtained for different parameter sets as a function of the applied magnetic field. As a reference, the radii obtained experimentally by Romming *et al*.^24^ are shown as green triangles. Note that we have obtained the skyrmion radii for the experimentally available field strengths by applying our definition of the skyrmion radius to the skyrmion profiles shown in Fig. 3(a) of ref.^24^. Antiskyrmions (ASk) were only metastable for fcc-Pd/Fe/Ir(111) with DFT parameters.

In the FM phase isolated skyrmions are metastable in our spin dynamics simulations up to a critical field for both hcp-Pd/Fe/Ir(111) and fcc-Pd/Fe/Ir(111). The skyrmion profiles were obtained by imposing the theoretical profile^46^ and relaxing this spin structure within our spin dynamics simulation. The radii were extracted as in ref.^46^. The obtained relaxed skyrmion profiles for both descriptions of the exchange are nearly indistinguishable as exemplified in the insets of Fig. 2. The resulting radii decrease rapidly with applied magnetic field (cf. Fig. 2) consistent with experiments^24^ and micromagnetic models^46^. Note that the range of metastable skyrmions stretches beyond B = 8 T for some cases as shown below.

For hcp stacking of Pd the radii are nearly the same using either *J*_eff_ or *J*_DFT_. However, isolated skyrmions are metastable for much larger fields if we use the full set of DFT parameters. For fcc stacking the radii are larger than for hcp stacking and they decrease faster with field considering *J*_eff_ leading to a crossing of the two curves. The radii obtained from experiments on Pd/Fe/Ir(111)^24^ are very close to those from *J*_DFT_ for fcc stacking of Pd. Antiskyrmions can be stabilized only if frustrated exchange is present as in fcc-Pd/Fe/Ir(111). Their radii are smaller than those of skyrmions in the same system. Below *B* = 3 T they become asymmetric, stretched objects and vanish for fields above *B* = 5.5 T.

Overall we find a very good agreement between simulations using the full set of exchange constants, the effective parameters and experimental data. We conclude that equilibrium properties of skyrmions in Pd/Fe/Ir(111) such as field dependent profiles or zero temperature phase diagrams can be accurately described by an effective (or micromagnetic) model for the exchange interactions.

### Skyrmion stability

**Figure 3:**
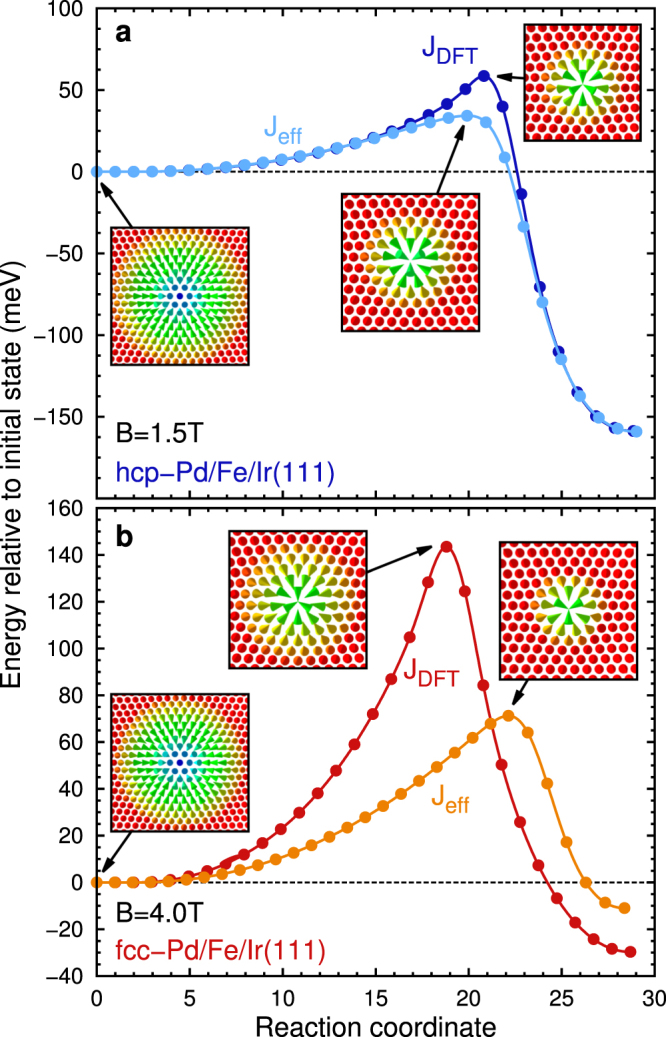
Minimum energy paths of skyrmion collapse. Energies of the spin configurations during a skyrmion collapse in (**a**) hcp-Pd/Fe/Ir(111) at *B* = 1.5 T and (**b**) fcc-Pd/Fe/Ir(111) at *B* = 4.0 T are shown over the reaction coordinate corresponding to the progress of the collapse. The energies are given with respect to the initial state, i.e. the isolated skyrmion. Simulations using both the effective exchange model (denoted by *J*_eff_) and taking all exchange constants from the DFT calculation into account (denoted by *J*_DFT_) are displayed. Insets show the spin structure of the initial and the saddle point configurations for both simulations.

Now we turn to the collapse of isolated skyrmions into the ferromagnetic state. The minimum energy path for this process is obtained using the GNEB method (see methods). Figure 3 shows the energy barriers calculated for both Pd stackings at selected field values and both types of describing the exchange. The initial skyrmion state and the mechanism of collapse are independent of how the exchange interaction is treated. The skyrmion shrinks along the path until at the saddle point the inner spins turn into the film plane after which the topological charge vanishes (see Supplementary Figs 1 and 2) and the skyrmion can collapse to the ferromagnetic state (see Supplementary Movies 1 to 4). This mechanism has already been described based on the effective exchange model^28–30,32,33^. However, the energy barriers are drastically enhanced if the frustrated exchange is explicitly treated in the simulations (see Supplementary Figs 1 and 2 for interaction resolved energy contributions along the reaction path). For the chosen magnetic field values we find an enhancement from about 34 to 59 meV for hcp-Pd/Fe/Ir(111) and from 71 to 143 meV for fcc-Pd/Fe/Ir(111).

**Figure 4:**
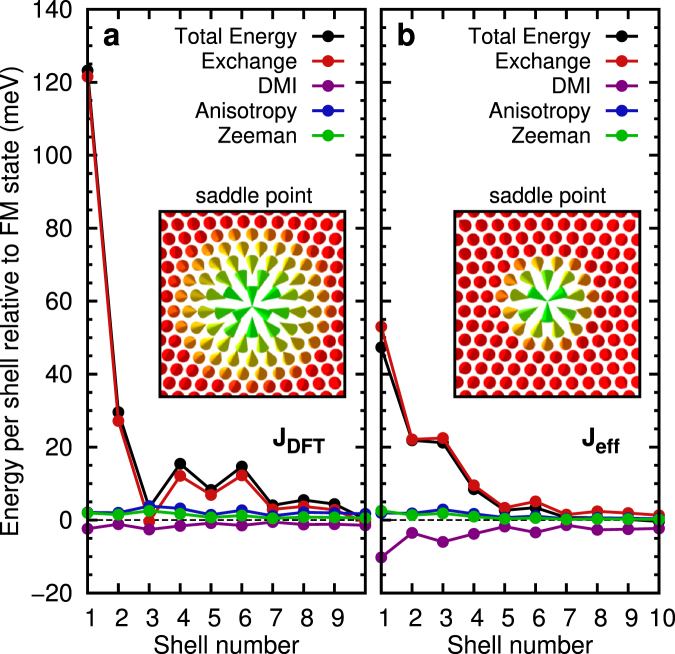
Energy contributions at the saddle point. Total energy per shell and contributions of the individual interactions are shown relative to the FM state for fcc-Pd/Fe/Ir(111) over the lattice shells of the saddle point for (**a**) DFT (*J*_DFT_) and (**b**) effective (*J*_eff_) parameters. Atoms with the same distance to the midpoint of the saddle point are defined as one shell. The insets show the corresponding saddle point configurations.

The spin structure at the saddle point is more extended if the full set of exchange constants is taken into account for fcc-Pd/Fe/Ir(111) as shown in the insets of Fig. 4. The main contribution to the energy at the saddle point with respect to the FM state stems from the exchange interactions while all other interactions are small. The inner shells of the spin structure give the largest contribution since the angles between adjacent spins are largest. This energy is much enhanced if the full set of exchange constants is considered since using an effective exchange *J*_eff_ underestimates this term. The role of the frustrated exchange is apparent also from its energy contribution along the reaction path (see supplementary Fig. S1) which exhibits a large barrier – an effect which is absent for an effective exchange. For hcp stacking of the Pd layer a similar picture is obtained (see Supplementary Figs 2 and 3).

**Figure 5:**
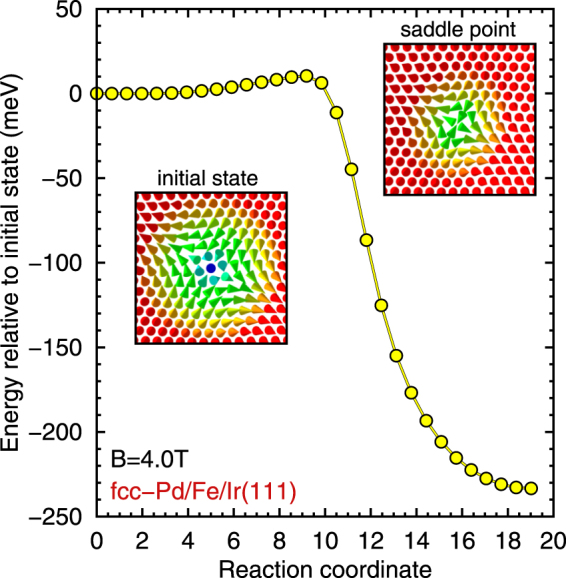
Minimum energy path of antiskyrmion collapse. Total energies of the spin configurations of an antiskyrmion collapse into the ferromagnetic state over the reaction coordinate corresponding to the progress of the collapse. The energies are given relative to the initial state, i.e. the isolated antiskyrmion. Insets show the isolated antiskyrmion and the saddle point configuration. In this simulation the full set of DFT parameters for fcc-Pd/Fe/Ir(111) were used.

Frustrated exchange interactions allow the appearance of metastable antiskyrmions^38^,^39,47,48^. We find non-vanishing energy barriers only in our simulations of fcc-Pd/Fe/Ir(111) using the full set of DFT parameters. In this case the exchange interactions alone already lead to a shallow energy minimum in the dispersion of spin spirals. The mechanism of antiskyrmion collapse is similar to that for skyrmions with a shrinking of diameter and a saddle point configuration with inner spins pointing in the film plane (see insets of Fig. 5 and Supplementary Movie 5). We obtain a small energy barrier of about 15 meV at a magnetic field of *B* = 4 T as shown in Fig. 5 (see Supplementary Fig. 4 for interaction resolved energy contributions along the path).

## Discussion

**Figure 6:**
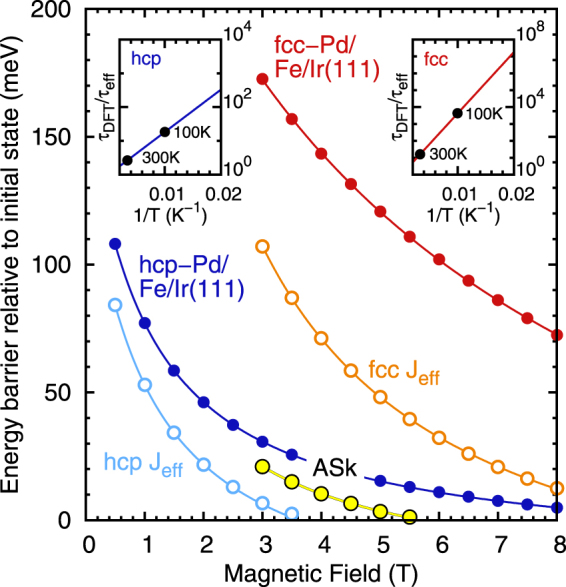
Energy barriers of skyrmion collapse vs. magnetic field and skyrmion lifetime enhancement. The energy barriers of isolated skyrmion collapse are shown as a function of applied magnetic field for the full set of DFT parameters as well as for the effective NN exchange model. The energy barriers of isolaed antiskyrmions which are metastable in fcc-Pd/Fe/Ir(111) using the DFT parameters are also displayed. The energy barriers are defined with respect to the initial states of isolated skyrmions or antiskyrmions. The inset shows the temperature dependence of the skyrmion lifetime enhancement *τ*_DFT_/*τ*_eff_ obtained from the energy barrier difference between DFT and effective parameters. Since the energy difference varies only by a few meV with field we take the values 72 meV for fcc (*B* = 4 T) and 25 meV for hcp (*B* = 1.5 T) stacking of Pd.

The energy barriers for skyrmion collapse exhibit a nonlinear dependence on the external magnetic field as shown in Fig. 6. For fcc-Pd/Fe/Ir(111) the barriers above the critical field of ${B}_{{\rm{c}}}^{{\rm{eff}}}\mathrm{=3.6}$ T are of the same order if we use *J*_eff_ as given by Hagemeister *et al*.^26^ based on Monte-Carlo simulations with an effective NN exchange. However, for all field values the barriers are drastically enhanced if the frustrated exchange in this system is treated properly. As a consequence isolated skyrmions are metastable up to much higher magnetic fields. Furthermore, antiskyrmions are only metastable if the full exchange interaction is taken into account with barriers on the order of 10 to 20 meV.

Even for hcp-Pd/Fe/Ir(111) which shows a ferromagnetic ground state at all field values there is a significant difference between the barriers for *J*_eff_ vs. *J*_DFT_. Despite the very similar zero temperature phase diagram (cf. Fig. 1b,c) isolated skyrmions are metastable at much larger fields for *J*_DFT_.

Within transition-state theory^49^ the lifetime *τ* of skyrmions depends exponentially on the energy barrier Δ*E*, i.e. $\tau ={\tau }_{0}\,\exp ({\rm{\Delta }}E/{k}_{{\rm{B}}}T)$ where *T* is the temperature and 1/*τ*_0_ is the attempt frequency. The enhancement of the lifetime due to taking exchange beyond NN into account for the barrier is ${\tau }_{{\rm{DFT}}}/{\tau }_{{\rm{eff}}}=\exp ([{\rm{\Delta }}{E}_{{\rm{D}}FT}-{\rm{\Delta }}{E}_{{\rm{eff}}}]/{k}_{{\rm{B}}}T)$. For both hcp and fcc stacking of Pd there is a huge enhancement by one or three orders of magnitude at 100 K, respectively (see insets of Fig. 6). Even at room temperature there is a large increase of skyrmion lifetime.

The key observation is that at the saddle point which defines the height of the energy barrier the angles between adjacent spins are large and the energy of this configuration cannot be accurately described here by the effective NN exchange or micromagnetic model. From the energy dispersion of spin spirals (cf. Fig. 1a) this is obvious since the effective NN exchange can only describe *E*(*q*) well close to the ferromagnetic state for small canting angles between adjacent spins while it breaks down at large *q* corresponding to large NN angles. The same logic dictates that the NN-exchange model should work better for the skyrmion annihilation at the boundary of a finite-size system since this process does not involve formation of singularities^50,51^. More detailed analysis of the applicability of the effective NN-exchange description of the skyrmion collapse at the boundary of a finite Pd/Fe/Ir(111) system as well as other itinerant electron magnets is, however, out of the scope of the present study and will be published elsewhere.

The effective exchange model even with parameters obtained from experiment^24,26,27^ is limited to describe skyrmion properties such as skyrmion profiles or zero temperature phase diagrams which rely on states close to the energy minimum. For all properties which require the treatment of configurations with large angles between adjacent spins such as skyrmion stability it is not sufficient. Therefore, properly treating exchange is also essential to simulate thermal fluctuations in order to obtain phase diagrams at finite temperature and phase transition temperatures^52^.

Our work shows the importance of accounting for the neighbor resolved exchange interaction even in systems with a ferromagnetic ground state and that engineering exchange frustration^45^ is a promising route towards enhanced skyrmion stability. Although we focus here on a particular system, the conclusions we arrive at are rather general and should be valid in other itinerant electron magnets where frustrated, long-range exchange is a typical feature.

## Methods

### First-principles calculations

We have explored the ultrathin film system Pd/Fe/Ir(111) from first-principles based on the full-potential linearized augmented plane wave method as implemented in the FLEUR code (www.flapw.de). Within this approach we can calculate the total energy of non-collinear magnetic structures such as spin spirals^40^ including the DMI in first order perturbation theory with respect to the spin-orbit coupling^41^. We have used a two-dimensional hexagonal p(1 × 1) unit cell within each layer and the in plane-lattice parameter of the Ir(111) surface as obtained from DFT in ref.^34^. The relaxed interlayer distances were also taken from ref.^34^. The magnetic properties were obtained within the local density approximation^53^ as previously^10,16,34^. All calculations have been carried out with a plane wave cutoff of *k*_max_ = 4.3 a.u.^−1^. The muffin tin radii were set to 2.23 a.u. for Fe and to 2.31 a.u. for Pd and Ir. We have used a 44 × 44 k-point mesh in the full two-dimensional Brillouin zone. In order to obtain quantitative parameters for the exchange interactions, we have converged the small energy minimum of the dispersion curve (Fig. 1(a)) with respect to the number of Ir substrate layers. With 5 layers of Ir substrate^34^ the energy minimum of fcc-Pd/Fe/Ir(111) is *E*_min_ = −1.1 meV/Fe (without spin-orbit coupling). Here, we have used asymmetric films with one layer Pd, one layer Fe and 15 layers of Ir substrate which leads to an energy minimum of *E*_min_ = −0.06 meV/Fe. To determine the exchange constants, we have used the energy dispersion curve of spin spirals *E*(*q*) for films with 15 Ir substrate layers for the spiral vector *q* in [0,1/4] and with 5 Ir layers for *q* in [1/4,2/3] (in units of 2*π*/*a*) along the $\bar{{\rm{\Gamma }}}\bar{{\rm{K}}}$ direction. The DM interaction has been taken from the calculations with 5 Ir substrate layers^34^ as it did not change with substrate thickness.

### Spin dynamics simulations

In order to calculate the energy differences between the FM, SkX and SS phases for the low temperature phase diagrams and to relax the isolated skyrmions and antiskyrmions, we used the Landau-Lifshitz equation of spin dynamics:

2 $$\hslash \frac{d{{\bf{m}}}_{i}}{dt}=\frac{\partial H}{\partial {{\bf{m}}}_{i}}\times {{\bf{m}}}_{i}-\alpha (\frac{\partial H}{\partial {{\bf{m}}}_{i}}\times {{\bf{m}}}_{i})\times {{\bf{m}}}_{i}$$

where *α* is the damping parameter, **m**_i_ is a single spin and *H* is the Hamiltonian given in Eq. (1). We have used damping parameters from *α* = 0.5 to *α* = 0.05 and time steps ranging from 0.05 fs to 0.1 fs to carefully relax the structures. The simulations are carried out on a timescale of 100 to 500 pico seconds and the equation of motion were solved with the semi-implicit integrator as proposed by Mentink *et al*.^54^.

### Geodesic nudged elastic band method

Minimum energy paths (MEPs) for skyrmion and antiskyrmion annihilation processes were identified using the geodesic nudged elastic band (GNEB) method^28^. The GNEB method involves taking some initially generated path between the energy minima, and systematically bringing that to the MEP. A path is represented by a discrete chain of states, or ‘images’, of the system, where the first one corresponds to the skyrmion or antiskyrmion configuration while the last one corresponds to the ferromagnetic state. At each image, the effective field is calculated and its component along a local tangent to the path is substituted by an artificial spring force between the images which ensures uniform distribution of the images along the path. This modified effective field is substituted in Eq. (2) in the overdamped regime and the whole chain of images evolved till convergence. The final, relaxed position of images gives discrete representation of the MEP. The energy maximum along the MEP corresponds to the saddle point (SP) on the multidimensional energy surface of the system and defines the energy barrier separating the stable states. In order to determine the maximum energy accurately, the highest energy image can be treated separately during the iterative optimization and made to move uphill in energy along the path. The effective field on this climbing image (CI) is calculated by deactivating the spring force acting on it and inverting the parallel component of the gradient field. After the CI-GNEB calculation has converged, the position of the CI coincides with the SP along the MEP and gives an accurate value of the SP energy.

### Data availability

The authors declare that the data supporting the findings of this study are available within the article and its Supplementary Information files.

## Electronic supplementary material

Supplementary Information

Supplementary Movie 1

Supplementary Movie 2

Supplementary Movie 3

Supplementary Movie 4

Supplementary Movie 5
