# Author Correction: Enhanced skyrmion stability due to exchange frustration

**DOI:** 10.1038/s41598-019-44360-7

**Published:** 2019-05-28

**Authors:** S. von Malottki, B. Dupé, P. F. Bessarab, A. Delin, S. Heinze

**Affiliations:** 1 Institute of Theoretical Physics and Astrophysics, University of Kiel, 24098 Kiel, Germany; 2 Institute of Physics, University of Mainz, 55128 Mainz, Germany; 3 School of Engineering and Natural Sciences - Science Institute, University of Iceland, 107 Reykjavik, Iceland; 4 University ITMO, St. Petersburg, 197101 Russia; 5 Department of Applied Physics, School of Engineering Sciences, KTH Royal Institute of Technology, Electrum 229, SE-16440 Kista, Sweden; 6 Department of Physics and Astronomy, Materials Theory Division, Uppsala University, Box 516, SE-75120 Uppsala, Sweden

Correction to: *Scientific Reports* 10.1038/s41598-017-12525-x, published online 26 September 2017

In Figure 2, the data points for the skyrmion and antiskyrmion radii (fcc J_DFT_, fcc J_eff_, hcp J_DFT_, hcp J_eff_ and ASk) were incorrect. Additionally, the legend of Figure 2,

“Skyrmion radius vs. magnetic field. Radii of skyrmions in Pd/Fe/Ir(111) obtained for different parameter sets as a function of the applied magnetic field. As a reference, the radii obtained experimentally by Romming *et al*.^24^ are shown as green triangles. Note that we have obtained the skyrmion radii for the experimentally available field strengths by applying our definition of the skyrmion radius to the skyrmion profiles shown in Fig. 3(a) of ref.^24^. Antiskyrmions (ASk) were only metastable for fcc-Pd/Fe/Ir(111) with DFT parameters.”

should read:

“Skyrmion radius vs. magnetic field. Radii of skyrmions in Pd/Fe/Ir(111) obtained for different parameter sets as a function of the applied magnetic field. As a reference, the radii obtained experimentally by Romming *et al*.^24^ are shown as green triangles. Note that we have obtained the skyrmion radii for the experimentally available field strengths by applying our definition of the skyrmion radius to the skyrmion profiles shown in Fig. 3(a) of ref.^24^. Antiskyrmions (ASk) were only metastable for fcc-Pd/Fe/Ir(111) with DFT parameters. Note that the radii obtained from experiments are very close to those from J_DFT_ for hcp stacking of Pd.”

The correct Figure 2 and its accompanying legend appear below as Figure 1. Note that the skyrmion profiles in the insets are the same as the profiles in Figure 2 of the original paper.

**Figure 1:**
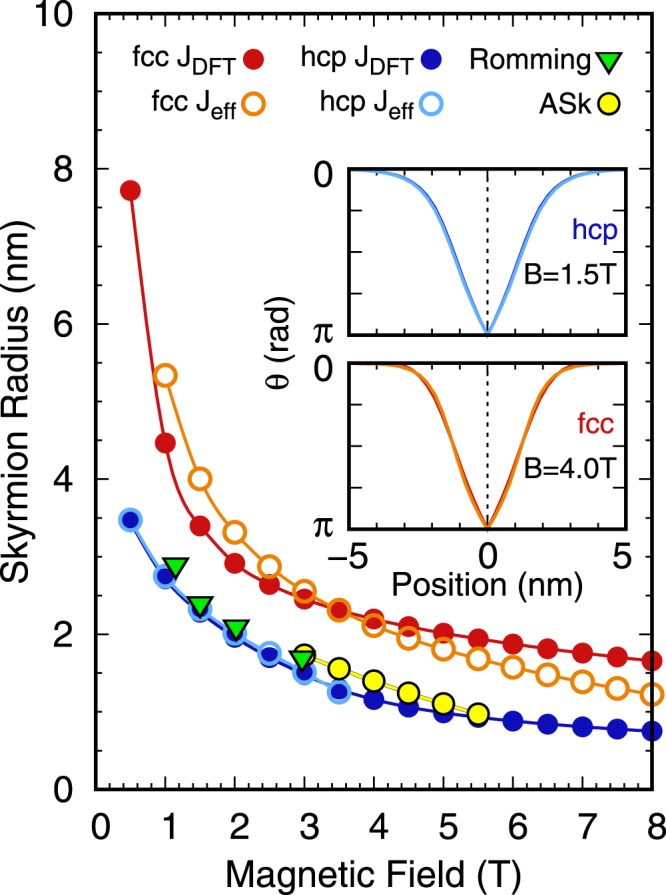
Skyrmion radius vs. magnetic field. Radii of skyrmions in Pd/Fe/Ir(111) obtained for different parameter sets as a function of the applied magnetic field. As a reference, the radii obtained experimentally by Romming *et al*.^24^ are shown as green triangles. Note that we have obtained the skyrmion radii for the experimentally available field strengths by applying our definition of the skyrmion radius to the skyrmion profiles shown in Fig. 3(a) of ref.^24^. Antiskyrmions (ASk) were only metastable for fcc-Pd/Fe/Ir(111) with DFT parameters. Note that the radii obtained from experiments are very close to those from J_DFT_ for hcp stacking of Pd.

The authors submitted these corrections 5 July 2018.

