# Supplementary material for: Enhanced skyrmion stability due to exchange frustration
Source: Sci Rep. 2017 Sep 26;7:12299. doi: 10.1038/s41598-017-12525-x (PMC5615047; doi:10.1038/s41598-017-12525-x)
Supplement: Supplementary file 1 — Supplementary Information [file 41598_2017_12525_MOESM1_ESM.pdf]

# Supplementary information for

## Enhanced skyrmion stability due to exchange frustration

S. von Malottki[1], B. Dupé[1,2], P. F. Bessarab[3,4], A. Delin[5,6], and S. Heinze[1]

[1]Institute of Theoretical Physics and Astrophysics, University of Kiel, 24098 Kiel, Germany

[2]Institute of Physics, University of Mainz, 55128 Mainz, Germany

[3]School of Engineering and Natural Sciences – Science Institute, University of Iceland, 107 Reykjavik, Iceland

[4]University ITMO, St. Petersburg, 197101, Russia

[5]Department of Applied Physics, School of Engineering Sciences, KTH Royal Institute of Technology, Electrum 229, SE-16440 Kista, Sweden

[6]Department of Physics and Astronomy, Materials Theory Division, Uppsala University, Box 516, SE-75120 Uppsala, Sweden

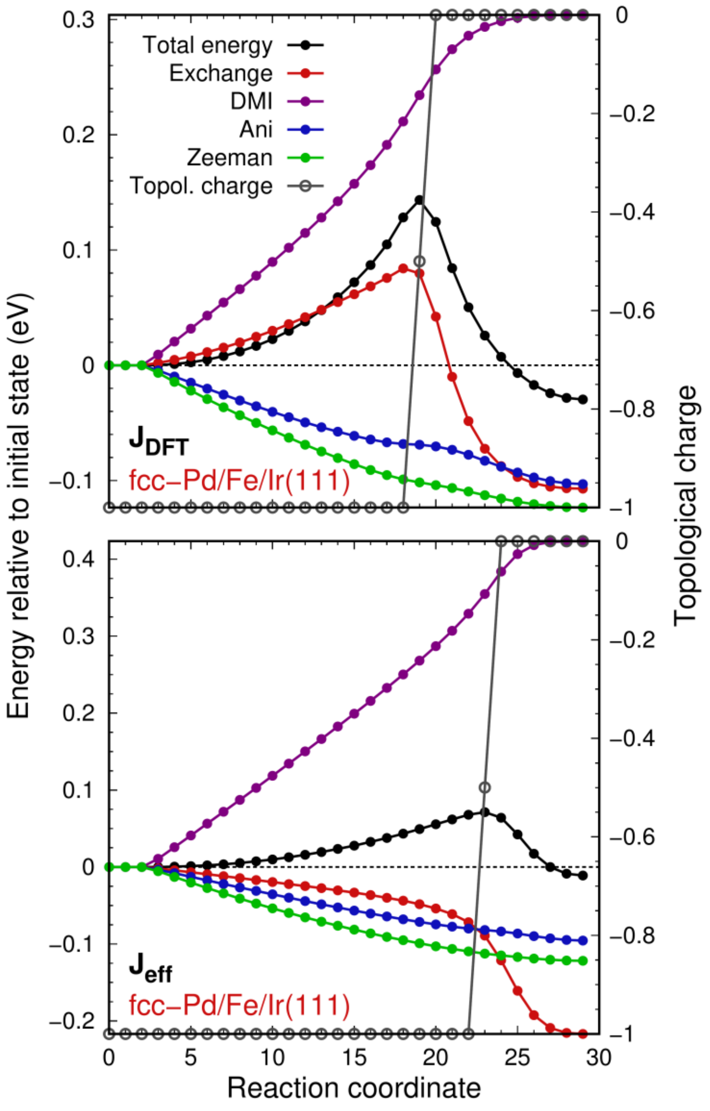


**Supplementary Figure 1 | Energy contributions and topological charge during skyrmion collapse in fcc-Pd/Fe/Ir(111).** Energy contributions from the different interactions (see legend, left axis) are shown versus the reaction coordinate from the initial (skyrmion) state to the final (ferromagnetic) state. The energies are summed over all atoms of the simulation box and are given relative to the energies of the initial isolated skyrmion state. The topological charge (open circles, right axis) is plotted versus reaction coordinate. The simulations are performed with the full set of DFT parameters for fcc-Pd/Fe/Ir(111) (upper panel, $J_{DFT}$) and with the effective nearest-neighbor exchange model (lower panel, $J_{eff}$). The magnetic field is B=4.0T.

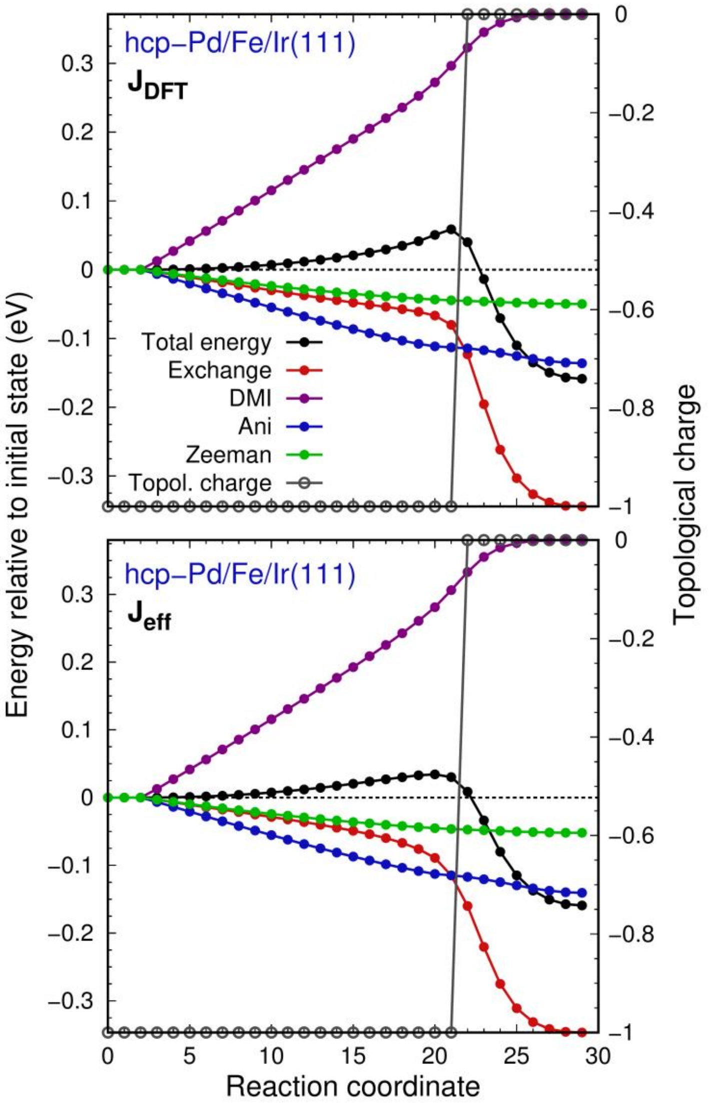


**Supplementary Figure 2 | Energy contributions and topological charge during skyrmion collapse in hcp-Pd/Fe/Ir(111).** Energy contributions from the different interactions (see legend, left axis) are shown versus the reaction coordinate from the initial (skyrmion) state to the final (ferromagnetic) state. The energies are summed over all atoms of the simulation box and are given relative to the energies of the initial isolated skyrmion state. The topological charge (open circles, right axis) is plotted versus reaction coordinate. The simulations are performed with the full set of DFT parameters for hcp-Pd/Fe/Ir(111) (upper panel, $J_{DFT}$) and with the effective nearest-neighbor exchange model (lower panel, $J_{eff}$). The magnetic field is B=1.5T.

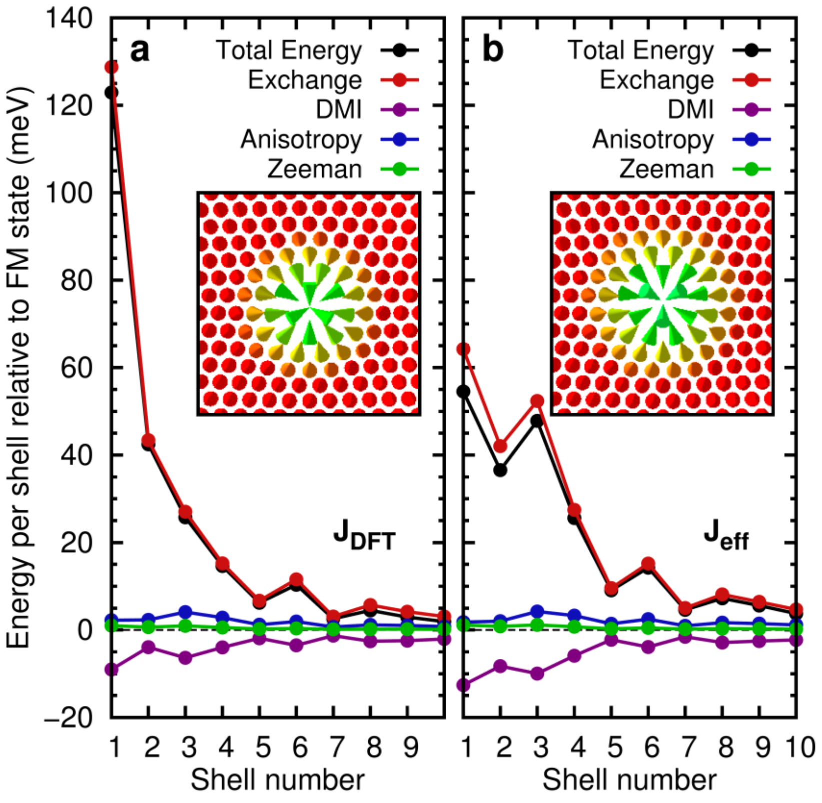


**Supplementary Figure 3 | Energy contributions at the saddle point for hcp-Pd/Fe/Ir(111).** Total energy per shell and contributions of the individual interactions are shown for hcp-Pd/Fe/Ir(111) over the lattice shells of the saddle point for (a) $J_{DFT}$ parameters and (b) $J_{eff}$ parameters. Atoms with the same distance to the midpoint of the transition state are defined as one shell. The insets show the corresponding saddle point configurations.

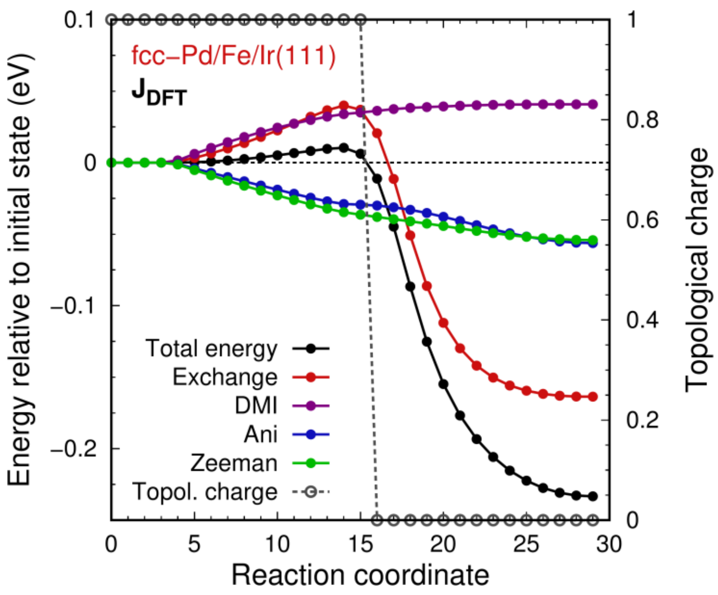


**Supplementary Figure 4 | Energy contributions and topological charge during antiskyrmion collapse in fcc-Pd/Fe/Ir(111).** Energy contributions from the different interactions (see legend, left axis) are shown versus the reaction coordinate from the initial (antiskyrmion) state to the final (ferromagnetic) state. The energies are summed over all atoms of the simulation box and are given relative to the energies of the initial isolated skyrmion state. The topological charge (open circles, right axis) is plotted versus reaction coordinate. The simulations are performed with the full set of DFT parameters for fcc-Pd/Fe/Ir(111) at B=4.0T.

**Supplementary Movie 1 | GNEB images of the skyrmion collapse for fcc-Pd/Fe/Ir(111) with $J_{DFT}$.** The MEP is shown on the left side of the movie, where the energy of the spin configuration during the skyrmion collapse is plotted over the reaction coordinate. Each point corresponds to an image of the GNEB calculation. The images are shown on the right side.

**Supplementary Movie 2 | GNEB images of the skyrmion collapse for fcc-Pd/Fe/Ir(111) with $J_{eff}$.** The MEP is shown on the left side of the movie, where the energy of the spin configuration during the skyrmion collapse is plotted over the reaction coordinate. Each point corresponds to an image of the GNEB calculation. The images are shown on the right side.

**Supplementary Movie 3 | GNEB images of the skyrmion collapse for hcp-Pd/Fe/Ir(111) with $J_{DFT}$.** The MEP is shown on the left side of the movie, where the energy of the spin configuration during the skyrmion collapse is plotted over the reaction coordinate. Each point corresponds to an image of the GNEB calculation. The images are shown on the right side.

**Supplementary Movie 4 | GNEB images of the skyrmion collapse for hcp-Pd/Fe/Ir(111) with $J_{eff}$.** The MEP is shown on the left side of the movie, where the energy of the spin configuration during the skyrmion collapse is plotted over the reaction coordinate. Each point corresponds to an image of the GNEB calculation. The images are shown on the right side.

**Supplementary Movie 5 | GNEB images of the antiskyrmion collapse for fcc-Pd/Fe/Ir(111) with $J_{DFT}$.** The MEP is shown on the left side of the movie, where the energy of the spin configuration during the skyrmion collapse is plotted over the reaction coordinate. Each point corresponds to an image of the GNEB calculation. The images are shown on the right side.
